# Enhanced Kinetic Removal of Ciprofloxacin onto Metal-Organic Frameworks by Sonication, Process Optimization and Metal Leaching Study

**DOI:** 10.3390/nano9101422

**Published:** 2019-10-08

**Authors:** Aliakbar Dehghan, Ali Akbar Mohammadi, Mahmood Yousefi, Ali Asghar Najafpoor, Mahmoud Shams, Shahabaldin Rezania

**Affiliations:** 1 Social Determinants of Health Research Center, Mashhad University of Medical Sciences, Mashhad 9138813944, Iran; DehghanAA@mums.ac.ir (A.D.); najafpooraa@mums.ac.ir (A.A.N.); 2 Department of Environmental Health Engineering, School of Health, Mashhad University of Medical Sciences, Mashhad 9138813944, Iran; 3 Department of Environmental Health Engineering, Neyshabur University of Medical Sciences, Neyshabur 9318614139, Iran; mohammadi.eng73@gmail.com; 4 Department of Environmental Health Engineering, School of Public Health, Iran University of Medical Sciences, Tehran 1449614535, Iran; Mahmood_yousefi70@yahoo.com; 5 Department of Environment & Energy, Sejong University, Seoul 05006, Korea

**Keywords:** metal organic frameworks (MOFs), adsorption, ciprofloxacin, sonication, metal leaching, kinetic

## Abstract

Metal-organic frameworks (MOFs) are currently recognized as unique platforms for environmental studies. This study evaluated the potential of nine MOFs from ZIF-8, ZIF-67, and UIO-66 families for the removal of ciprofloxacin (CIP), a toxic, bio-accumulative, and persistent fluoroquinolone antibiotic. ZIF-67-SO_4_, with a rhombic crystalline morphology and 1375 m^2^/g BET surface area, has the highest CIP adsorption efficiency among the studied MOFs. The mathematical sorption model predicted that the highest CIP removal (99.2%) occurs when adsorbent dose, pH, and agitation time are adjusted to 6.82, 832.4 mg/L, and 39.95 min, respectively. Further studies revealed that the CIP adsorbed onto ZIF-67-SO_4_ in monolayer (q_max_: 2537.5 mg/g) and chemisorption controlled the rate of the process. Mass transfer kinetic coefficients improved significantly by sonication at 35 KHz in comparison with mechanical agitation. Thermodynamic parameters (minus signs of ∆G° [7.8 to 14.2], positive signs of ∆H° (58.9 KJ/mol), and ∆S° (0.23 KJ/mol·K)) demonstrated the spontaneous, endothermic, and chemical sorption of CIP. The level of cobalt leached from ZIF-67-SO_4_ structure varied 1.2–4.5 mg/L, depending on pH, mixing time, and agitation type. In conclusion, the excellent adsorption properties of ZIF-67-SO_4_ for CIP, made it an outstanding candidate for environmental protection purposes.

## 1. Introduction

Metal-organic frameworks (MOFs) are an emerging class of nanoporous structures that composed of organic units (linker or ligand) and inorganic metallic units with one, two, or three dimensions [1,2]. Tunable and regular porosity, various possible synthesis routes, and the capability of structural engineering and post-synthesis modifications, make these hybrid crystalline materials a platform for sensing, drug delivery, gas storage and purification, luminescence, catalyst, and adsorption [2,3].

Currently, adsorption is extensively used for the removal of pollutants from aqueous systems [4]. To overcome the drawbacks of ordinary adsorbents, such as slow adsorption rate and low capacity, researchers have focused extensively on novel adsorbents [4]. With a very high surface area, excellent porosity, and voids fraction, MOFs provide a promising perspective in sorption field. Until recently, MOFs were applied for the sorption of arsenic [5], fluoride [6], dyes [7,8,9], among others [10,11].

Zeolitic imidazolate frameworks (ZIFs) are a class of MOFs composed of cobalt or zinc metal ions and organic imidazole linkers. They are highly stable in aqueous and harsh environments. ZIF-8 is a nitrogen-coordinated ZIF that consisting of zinc (II) ion and 2-methylimidazole linker to build tetrahedral units. It is known for its superior advantages in membrane separation, adsorption, and catalysis. ZIF-67 has the same sodalite zeolite-type topology as ZIF-8 with cobalt ions instead of zinc [12]. 

UIO-66 is a zirconium base MOF with hexa-nuclear zirconium clusters connected by terephthalate linkers. Owing to its stable structures under harsh thermal and chemical environments, UIO-66 has received considerable attention for the purpose of different environmental applications [1].

Ciprofloxacin (CIP) is a member of fluoroquinolone antibiotics that have diverse medical purposes. CIP is a mobile, soluble, and non-biodegradable contaminant that could not be removed completely during the conventional wastewater treatment system. Due to their toxic, bio-accumulative, and persistent nature, the presence of CIP and its residues in water bodies pose a major concern to the environment and human health [3,4].

No study has yet examined the CIP removal by ZIFs or UIO-66. Thus, we studied the feasibility of CIP removal under various key operating parameters, named pH, antibiotic concentration, MOFs dosage and contact time. A mathematical process model was developed and optimized to maximize CIP removal efficiency. Kinetic coefficients for CIP adsorption under conventional mechanical stirring and ultra-sonication provided. The work also covers the study of parameters that controls the leak of heavy metal from the MOF structure.

## 2. Experimental Design, Materials, and Methods

### 2.1. Reagents and Chemicals

All the chemical used in this study were prepared from Sigma Aldrich (St. Louis, Mo, USA) and Merck (KGaA, Darmstadt, Germany) and used without modification. The chemical structure of CIP and its physiochemical properties are presented in Table 1. 

### 2.2. Synthesis of Metal-Organic Frameworks (MOFs)

ZIF-8, ZIF-67, and UIO-66 were prepared according to the procedures described in the supplementary file. Different crystal topologies for ZIF-8 and ZIF-67 were obtained by changing the mole ratios and metal compounds in synthesis method as summarized in Table 2. The resulting product in the synthesis solution, (white in the case of ZIF-8, purple for ZIF-67, and yellow for UIO-66) was centrifuged at 3000 rpm for 10 min and washed several times with deionized water. The residual solid was oven-dried at 70 °C to remove any remaining moisture in the MOF pores [14,15,16].

A batch experimental system was employed in this study and the experiments were carried out in 25 °C and agitated at a speed of 200 rpm and duplicate (mean values). The pH of the CIP solution was adjusted to the required values using dilute NaOH or HCl solutions. The experiments were conducted by changing the variables of pH (4–12), initial CIP concentration (10–100 mg/L), adsorbent dosage (0.2–1 g), and contact time (10–90 min). The residual concentration of CIP in the solution was determined using high liquid performance chromatography (HPLC, knauer, smartline, Germany) equipped with a vortex column and UV detector at a flow rate of 0.8 mL min^-1^ and wavelength 270 nm.

### 2.3. Experimental Design

Response surface methodology (RSM) is a collection of statistical and mathematical techniques and also one of the ideal tools for experimental design optimization where several variables affect the response of interest. It is an economical approach to determine the maximum efficiency in a shorter period of time and with conducting the least number of experiment runs [17,18].

The effect of four independent variables was investigated using the central composite design (CCD) with a total 30 runs consisting of 2 × 4 = 8 axial points, 2^4^ = 16 factorial points, and six center points. To explain functional interactions between input parameters and the response, a second-order polynomial equation was applied to model the sorption process as following:

(1) $$Y=\beta_{0}+\overset{k}{\underset{i=1}{\sum }}\beta_{i}X_{i}+\overset{k}{\underset{i=1}{\sum }}\beta_{ii}X_{i}^{2}+\overset{k-1}{\underset{i=1}{\sum }}\overset{k}{\underset{j-1}{\sum }}\beta_{ij}X_{i}X_{j}+\varepsilon \;$$

where Y is the response, X_i_ and X_j_ are the independent variables, β_0_ is a constant value, β_i_, β_ii_, and β_ij_ are the regression coefficients for a linear, second-order, and interaction effects, respectively. ε is the error of the model [17,18].

In the first phase of this study, the synthesized MOFs were used at 15–30 min contact time to find the adsorbent with the highest affinity to the CIP. The amount of CIP removal and the amount of CIP uptake (q_e_) at the equilibrium were calculated by using the following equations, respectively:

(2) $$\%\;\mathrm{Removal}=\frac{\;\left(C_{0}-\mathrm{Ce}\right)\times 100}{C_{0}}$$

(3) $$\mathrm{qe}=\frac{\;\left(C_{0}-\mathrm{Ce}\right)\times V}{W}$$

where $C_{0}$ is initial CIP concentration, Ce is final CIP concentration, V is the volume of the solution (L), and W is the mass of the MOF (g) used in the experiments. During the first phase of study, the CIP detaches from the MOF surfaces gradually as time increases beyond about 30 min. On the basis of these observations, the upper limit of time in designing the experiments was considered to be 30 min. The study also revealed that ZIF-67-SO_4_ has the highest CIP removal. Thus, ZIF-67-SO_4_ was selected to continue the CIP adsorption study. Response surface methodology (RSM) using R software was utilized to model the sorption process. Before run design, the operational variables were coded in the following order: X_1_, X_2_, X_3_, and X_4_ representing contact time (min), adsorbent dosage (g/L), pH and CIP concentration (mg/L), respectively. The range of independent variables and their coded values are shown in Table 3.

A total of 29 experiments with four replicates at the center point were performed in a Box-Behnken design (BBD) design. The coded values ($x_{i}$) of the input variables were determined by the following equation [19]:

(4) $$x_{i}=\frac{X_{i}-X_{0}}{∆X}\;$$

where X_i_ is the actual value of input variables, X_0_ is the actual value of input variables at the center point, and ∆X is the step change value. After performing the experiments according to the design matrix (Table 4), the data were analyzed using the analysis of variance (ANOVA), coefficient of determination (R^2^), and lack of fit (LOF).

## 3. Results and Discussion 

### 3.1. Adsorbent Characterization

**Figure 1:**
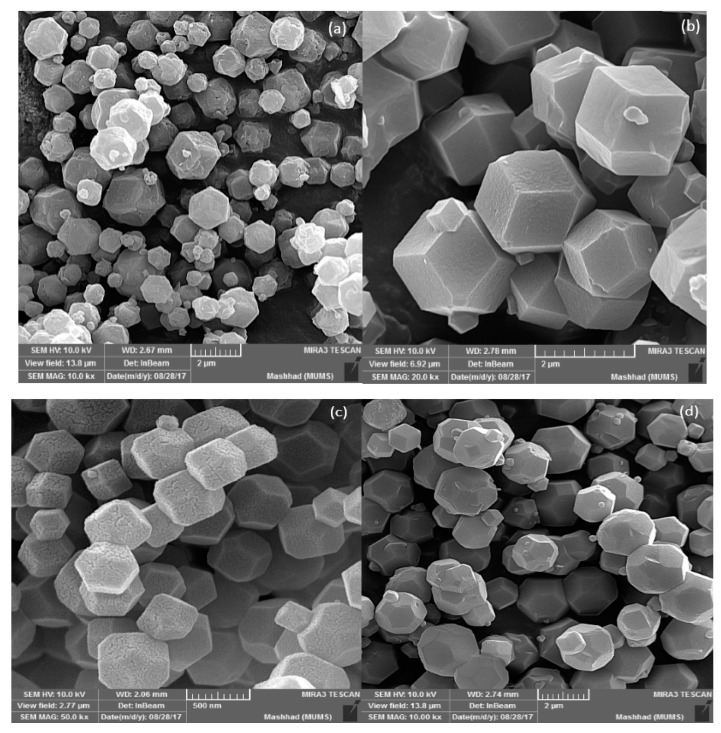

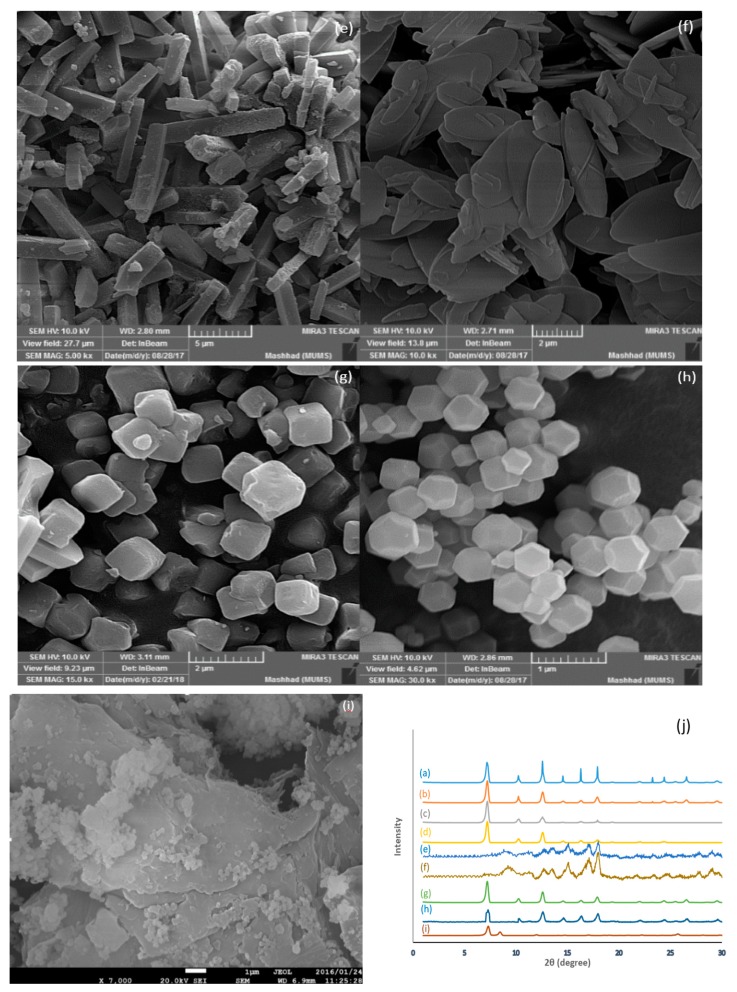
Characteristics of the as-synthesized MOFs: scanning electron microscope (SEM) image of MOFs; (**a**) ZIF-67-OAC, (**b**) ZIF-67-Cl, (**c**) ZIF-67-NO_3_, (**d**) ZIF-67-SO_4_, (**e**) ZIF-8-Cuboid, (**f**) ZIF-8-Leaf, (**g**) ZIF-8-Cube, (**h**) ZIF-8-Octahedron, (**i**) UIO-66, (**j**) x-ray diffraction (XRD) pattern.

The adsorbents were characterized by x-ray diffraction (XRD, Unisantis S.A, XMD300 model, Geneva, Switzerland) and field emission scanning electron microscope (FE-SEM, MIRA3 TESCAN, Czech Republic) analysis. Figure 1 shows the XRD pattern of the MOFs. The conformity of XRD peaks for MOFs with those reported in the literature shows their pure crystalline structures [14,15,16]. Moreover, the uniformity of micrometer-sized crystals and geometrical structure of the particles in SEM analysis, as shown in Figure 1, confirms the preciseness of the MOFs’ synthesis.

### 3.2. Study of MOFs for Ciprofloxacin (CIP) Removal

**Figure 2:**
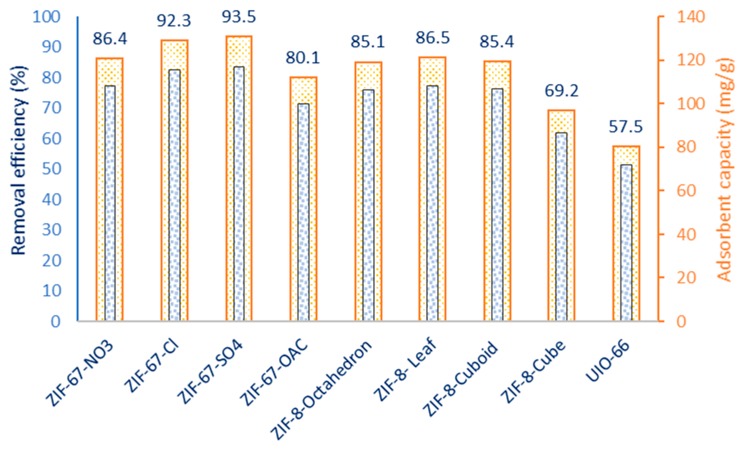
Removal efficiency and adsorption capacity of as-synthesized MOFs for CIP (CIP: 62.5 mg/L, MOF: 0.5 g/L, time: 30 min).

The affinity of as-synthesized MOFs for CIP was evaluated in a batch system by observing their removal efficiency in the presence of a fixed 500 mg MOF per L. Furthermore, the capacity of adsorbents for CIP in the equilibrium, after 30 min, were determined. The results presented in Figure 2 show that the CIP removal was highest for ZIF-67-SO_4_, and the level of MOF affinity decreased by ZIF-67-Cl > ZIF-8-NO_3_ > ZIF-8 leaf = ZIF-8 octahedron > ZIF-8-OAc > ZIF-8-cube > UIO-66. ZIF-67-SO_4_ also shows the highest capacity for CIP in this stage.

### 3.3. Model Development Using Response Surface Methodology (RSM)

To evaluate the suitability of linear, interaction (2Fl), quadratic and cubic models, the statistical indicators from the sequential model were compared, as seen in Table S1 in Supplementary Materials. The simplest model which also featured an acceptable statistical fitness was recognized as the best model. The larger amount of F-value and smaller p-value coefficients in Table S1 demonstrate the quadratic model as the best fit. 

ANOVA could explain how the change in independent variables influences the response values. The results of ANOVA are given in Table S2. For the model, both R^2^ (0.97) and adjusted R^2^ (0.95) are close to one and within ± 0.2 range of each other. Moreover, the F-value (41.85), P-value (5.718 × 10^–9^), and non-significant value for lack of fit (0.1155) indicate that the model is statistically adequate. 

**Figure 3:**
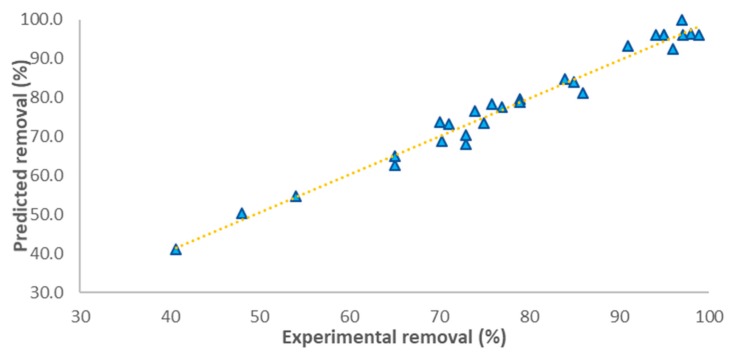
Experimental versus predicted removal values for CIP adsorption onto ZIF-67-SO_4_.

The preciseness of the model for predicting the CIP removal under experimental conditions is apparent from the uniform distribution of experimental points close to the regression line as seen in Figure 3.

According to the coefficients obtained for each term presented in Table 5, the following equation was developed for the prediction of CIP removal values:

(5) $$\begin{array}{ll} \mathrm{CIP}\;\mathrm{removal}\;\left(\%\right) & =\;96.02+9.18{\;X}_{1}+\;3.42{\;X}_{2}-\;9.03{\;X}_{3}\;-6.38{\;X}_{4}-\;3{\;X}_{1}X_{2}\\ & +7.08{\;X}_{1}X_{3}-1.63{\;X}_{1}X_{4}+0.5{\;X}_{2}X_{3}-2.25{\;X}_{2}X_{4}-X_{3}X_{4}-\;6.82\;X_{1}^{2}\\ & -5.42\;X_{2}^{2}\;-22.83\;X_{3}^{2}\;-6.41\;X_{4}^{2} \end{array}$$

In the above quadratic model, the level of impaction for each individual variable and their interactions on CIP removal is attainable. The positive and negative sign of terms in the model demonstrates that the adsorption increased and decreased by variable value, respectively [13].

### 3.4. Effects of Model Variables and Their Interactions

**Figure 4:**
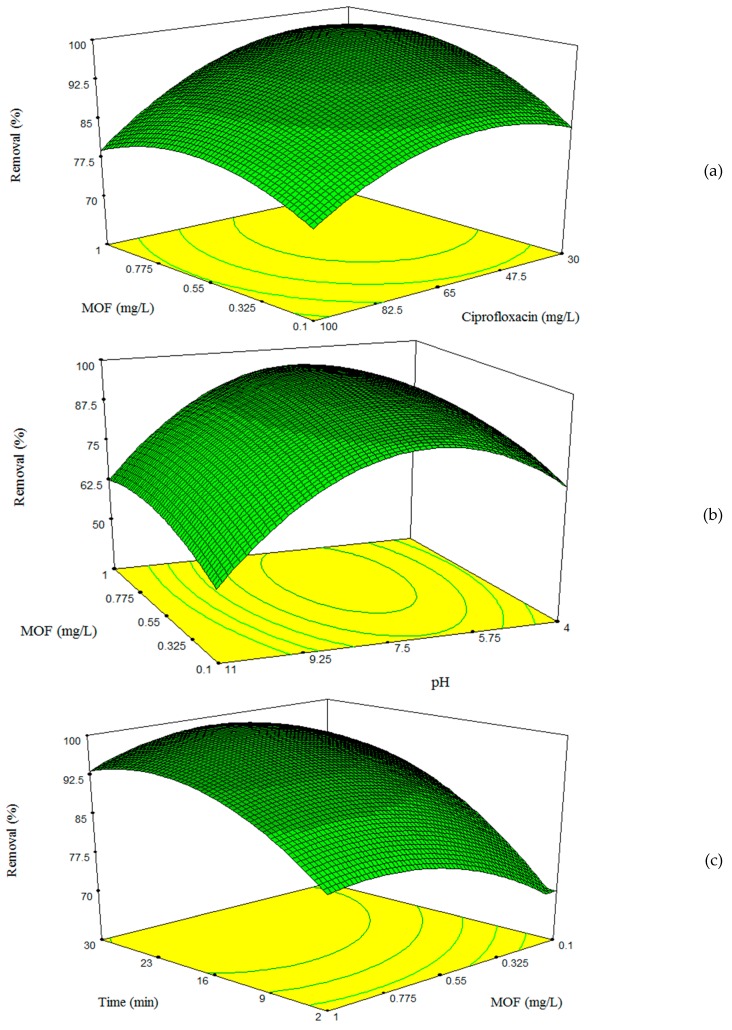
Effect of sorption variables on CIP removal: (**a**) the effect of MOF and CIP concentration, (**b**) MOF and pH, (**c**) MOF and agitation time.

A solid-to-solution ratio is one of the main factors in a batch adsorption process to determine the efficiency of an adsorbent [3,20]. The effect of MOF dosage on removal efficiency of CIP is displayed in Figure 4a. With increasing MOF dosage from 0.1 to 0.55 g/L, firstly, removal of CIP increased in all of the initial concentration of CIP because of the more available active sites on the adsorbent; however, when MOF dosage was more than 0.55 g/L, a decrease was observed, which can be related to the increase in the pH of the solution by MOF dose. 

Solution pH can change the surface speciation of both the adsorbate and adsorbent. It is necessary to explore the effect of solution pH on removal efficiency to understand the mechanism of adsorption between them [20]. The interaction plot of pH and MOF dosage is illustrated in Figure 4b. When the pH of the solution increases from 3 to 7.5, adsorption of CIP on MOF also increased. However, at pH values above 7.5, it decreased. The root of this adsorption behavior can be attributed to protonation–deprotonation reactions in groups of CIP molecule including cationic species (pH < 5.9), zwitterionic species (5.9 < pH < 8.9), or anionic species (pH > 8.9), as shown in Table 1. In previous studies, the point of zero charges of MOF was found to be at 8.7 [21], showing a positively charged surface at pH < 8.7 and negatively charged surface of ZIF-67 at pH > 8.7 [21]. As shown in Figure 4c, removal percentage increased when the CIP molecules had forms of cationic or zwitterionic species, likely a result of electrostatic attraction between more numbers of negatively charges on the surface of CIP and positively charged surface of the adsorbent. While in the presence of anionic species of CIP, the surface of CIP and MOF were both negative, leading to repulsion between them and decreasing removal efficiency [3,22]. Maximum adsorption of CIP was obtained at pH 7.3, which was selected for further study. 

### 3.5. Model Optimization and Adequacy Checking 

Optimization is the final goal in modeling a sorption process that provides the condition in which the sorption proceed under highest efficiency. By using Equation (5), the optimum values for the operating variables in the current study were achieved and are presented in Table 6. Confirmatory tests were conducted by simulating the optimum conditions and subsequently, the obtained CIP removal value was compared with those predicted by the model. 

### 3.6. Isotherm Modeling

Adsorption time was prolonged to 8 h for determining the equilibrium time of adsorbent. Five two-parameter and six three-parameter isotherm models were employed to fit the experimental data. Nonlinear form of isotherm models was used for data fitting duo to lack of ability of linear regression to describe experimental data in isotherm models with more than two parameters. Table S3 in the supplementary file shows the list of nonlinear isotherm models [23,24]. Table 7 shows the parameters of isotherm models.

**Figure 5:**
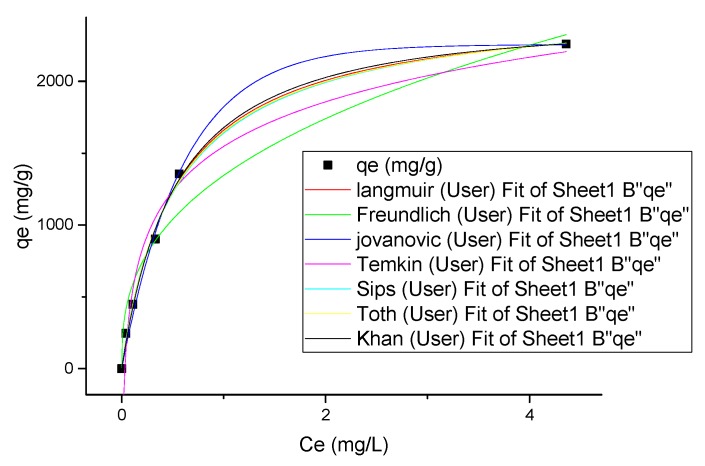
Nonlinear plots of isotherm models used for adsorption of CIP.

Nonlinear plots of the selected isotherm models are shown in Figure 5. As can be seen, both of two-parameter and three-parameter isotherm models are capable of fitting the experimental data. However, among the selected isotherm models, the Langmuir model could describe isotherm data better than other models because of the higher $R_{\mathrm{Adj}}^{2}$. The maximum adsorption capacity of the adsorbent was obtained to be 2.537 g/g according to Langmuir isotherm model, which is close to those suggested by Jovanovic (2.256 g/g), Sips (2.593 g/g), and Khan (2.772 g/g) isotherms. The sum of the square errors (SSE), adjusted linear coefficient of determination (R^2^_Adj_), and Chi-square (x^2^) tests were used to check and compare the validity of the models. The ability of the model to predict experimental data could be concluded from the lower values of SSE and x^2^ and higher values of R^2^ [25]. As a result, Langmuir isotherm was selected as the best model for fitting data due to the lower SSE and x^2^ and higher R^2^ values.

### 3.7. Kinetics Modeling under Convectional Mixing and Sonication

**Figure 6:**
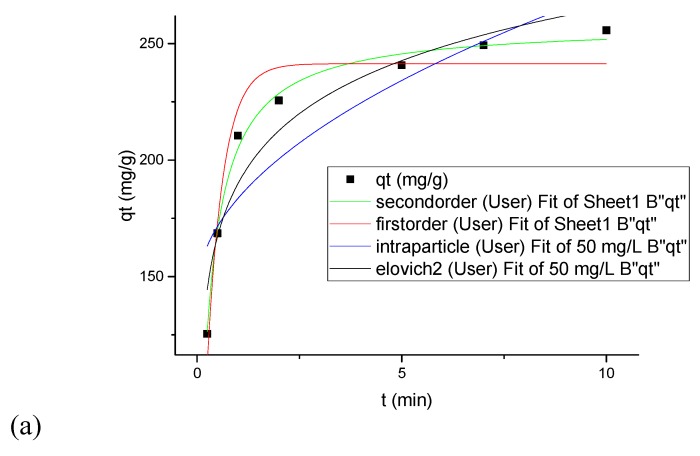

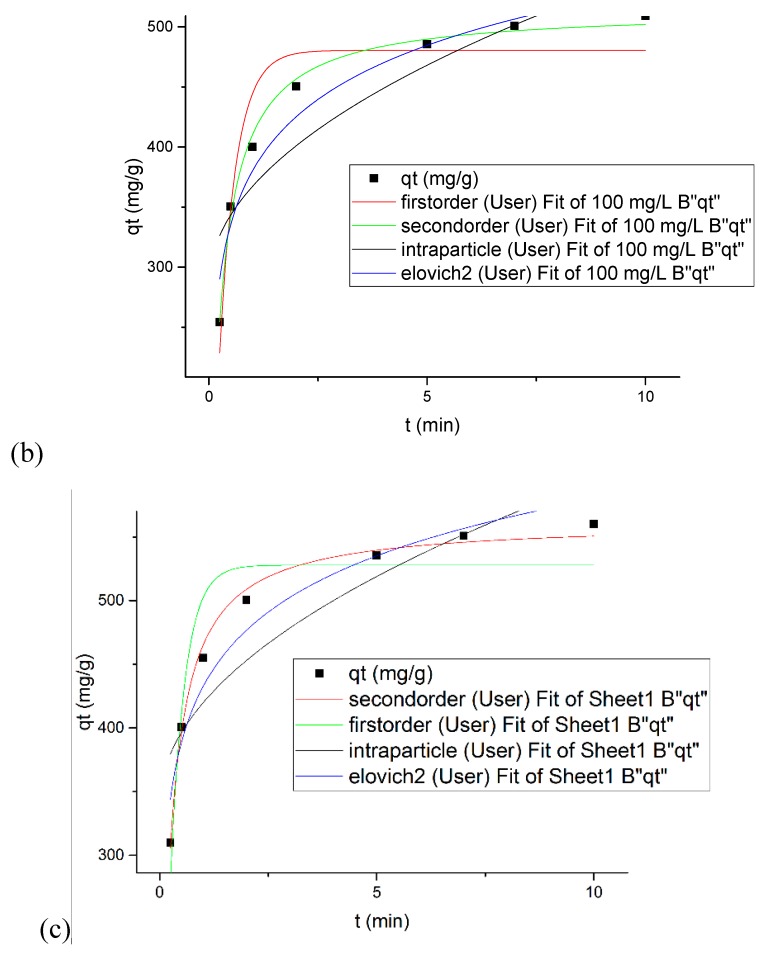
Nonlinear plots of kinetic models used for adsorption of CIP (**a**) 50 mg/L, (**b**) 100 mg/ L, (**c**) 100 mg/L sonication.

Kinetic parameter values of CIP adsorption on ZIF under mechanical mixing and sonication are reported in Table 8. Fitting plots of CIP adsorption capacity (q_t_) of ZIF against time (t) are shown in Figure 6. Pseudo-second-order model was found to be the best fitting model because it provided the highest determination coefficients (R^2^ ≥ 0.992) in comparison with the others in all of CIP initial concentration. In addition, the maximum adsorption capacity of ZIF predicted by the pseudo-second-order model (514.8 mg/g) is closer to experimental ones (509.06 mg/g) when compared with those calculated by other models. Rate constant values of pseudo-second-order model (k_2_) decreased with an increasing initial concentration of CIP, suggesting that the adsorption rate decreased as CIP concentration increased [21]. 

The effect of mechanical mixing and sonication on CIP adsorption was studied by performing the kinetic experiments under the magnetic string at an initial concentration of CIP 50 and 100 mg·L^−1^ and in an ultrasonic bath at an initial concentration of CIP 100 mg·L^−1^. As seen in Table 8, experimental q_e_ and rate constant of the pseudo-second-order model in the ultrasonic bath were higher than those reported under mechanical mixing. The formation of micro jets and high-pressure zones during the sonication could promote the migration of CIP molecules into the nano-sized pores of ZIF-67-SO_4_. Similar findings were also obtained for the removal of blueberry anthocyanins [26], congo red [27], phosphate [28], and different dyes [29]. Studies also demonstrated that sonication can be used as a modifier to promote the structural properties of adsorbents to reach higher capacity for target contaminants [30,31].

### 3.8. Thermodynamic of Adsorption 

All the chemical, physical, and biological processes could be influenced by the temperature of the environment in which they occur. The thermodynamic study aimed to investigate the feasibility of process progressiveness by studying changes in environmental temperature. Thermodynamic parameters also reveal the nature of adsorption in term of physisorption or chemo-sorption. The description of a sorption behavior in a thermodynamic study could be done by using the most widely used standard enthalpy (∆H°), standard entropy (∆S°), and Gibb’s free energy (∆G°) which are obtainable using the Van’t Hoff plot (Ln K_0_ vs 1/T) according to the following:

(6) $$∆G{}^{\circ}=-RT\;lnK_{L}\;$$

(7) $$lnK_{L}=\frac{\Delta S}{R}-\frac{∆H{}^{\circ}}{RT}$$

where R and T are universal gas constant (8.314 J/mol·K) and temperature (K), respectively. Thermodynamic parameters for CIP removal under different temperature are presented in Table 9. The negative values for ∆G° reveal the spontaneous nature of adsorption. Decreasing the CIP removal by temperature and the positive sign of ∆H° and ∆S° present the endothermic nature of adsorption. Furthermore, the absolute ∆G° in the table is higher than 40 kJ/mol, indicating that chemisorption is the predominant mechanism for the CIP adsorption [32,33].

### 3.9. ZIF-67-SO_4_ Structural Stability

**Figure 7:**
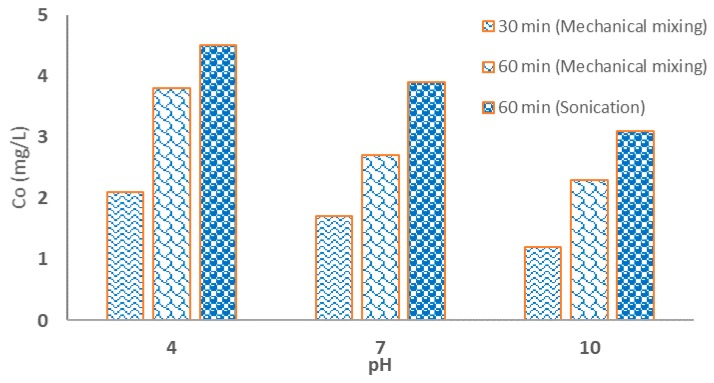
Leaching of Co from ZIF-67-SO_4_ structure as a function of time, pH, and mixing type.

Due to the presence of cobalt in the structure of ZIF-67, levels of this heavy metal may be present in treated waters. Thus, it is crucial to investigate the level of cobalt leach in the solution. Cobalt concentration in the treated water also could provide an indirect indication for the structural stability of ZIF-67-SO_4_ under environmental agitation. The stability test was conducted under different initial pH for 30 and 60 min under conventional mechanical agitation and sonication at 35 KHz. Figure 7 shows the concentration of Co ions in the final solution when ZIF-67-SO_4_ was applied to the solutions. As expected, the concentration of Co ions increased dramatically by increasing the corrosiveness as a result of higher H^+^ in low pH. Our previously published work showed that ZIFs were completely dissolved in strong acidic environments (with a pH of about 3) [28]. Moreover, the concentration of Co ions increases by mixing time (~30–60 min), which may be attributed to an increased opportunity for metal ions to leach from the ZIF-67-SO_4_ structure. As Figure 7 shows, sonication could result in much higher Co ions when compared with conventional mixing. This could be attributed to higher shear forces during sonication which results in de-agglomeration of crystals and the probable break-down in the structure of ZIF-67-SO_4_ [26,27]. 

## 4. Conclusions

Environmental pollution with antibiotics poses great concerns to both humans and the ecosystem. Control of ciprofloxacin as a toxic, bio-accumulative, and persistent antibiotic is crucial for the pharmaceutical industry. Herein, the potential of nine members of MOFs for ciprofloxacin (CIP) removal was investigated and observed to be in the order of ZIF-67-SO_4_ ˃ ZIF-67-Cl > ZIF-8-NO_3_ > ZIF-8 leaf > ZIF-8 octahedron > ZIF-8-OAc > ZIF-8-cube > and UIO-66. A prediction model for CIP removal was developed by performing the experiments according to BBD. Optimization of the model yield the highest CIP removal (99.2%) to occur at 6.82, 832.4 mg/L, and 39.95 min for adsorbent dose, pH, and agitation time, respectively. Among nonlinear form of two-parameter and three-parameter isotherm models, the Langmuir model described the data well according to SSE, R^2^_Adj_, and x^2^. The maximum adsorption capacity of the adsorbent was 2.537 g/g according to the Langmuir model. Kinetic constants were improved in the sonication at 35 KHz when compared with mechanical agitation mode, and in both cases followed the pseudo-second-order model. The level of cobalt leached from ZIF-67-SO_4_ was influenced by solution pH, mixing time, agitation type, and was observed to be in the range of 1.2–4.5 mg/L. Thermodynamic parameters (minus signs of ∆G° (7.8 to 14.2), positive signs of ∆H° (58.9 KJ/mol), and ∆S° (0.23 KJ/mol·K)) demonstrate the spontaneous, endothermic, and chemisorption nature of the process. In short, ZIF-67-SO_4_ shows outstanding capacity and excellent affinity toward CIP and thus is a promising candidate for preventing CIP discharge into the aquatic environment.

## Figures and Tables

**Table 1:** Structural and chemical properties of ciprofloxacin and its pKa [3,13].

Ciprofloxacin Structure	Molecular Formula	pKa
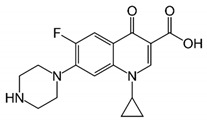	C_17_H_18_FN_3_O_3_	pKa_1_ = 5.9
		pKa_2_ = 8.9

**Table 2:** Summary of synthesis condition and structural properties of as-synthesized metal-organic frameworks (MOFs).

MOFs	Ligand	Metal Source	Ligand/ Metal Mole Ratio	Structural Morphology	BET Surface Area (m^2^/g)	Total Pore Volume (cm^3^/g)
UIO-66	Terephthalic acid	ZrCl_4_	1	Plate	765	0.44
ZIF-67	2-methylimidazole	Co(NO_3_)_2_	20	Granular	734	0.34
	2-methylimidazole	Co(OAC)_2_	20	Rhombic Dodecahedron	1323	0.57
	2-methylimidazole	CoSO_4_	20	Rhombic Dodecahedron	1375	0.62
	2-methylimidazole	CoCl_2_	20	Rhombic Dodecahedron	1278	0.52
ZIF-8	2-methylimidazole	Zn(NO_3_)_2_	29.4	Octahedron	1151.2	0.58
	2-methylimidazole	Zn(OAc)_2_	7.9	Leaf	12.7	0.04
	2-methylimidazole	Zn(NO_3_)_2_	7.9	Cuboid	890.4	0.48
	2-methylimidazole	Zn(NO_3_)_2_	2	Cube	978	0.51

**Table 3:** Experimental range and levels of independent variables.

Factor	Variable Level			
	Code	−1	0	+1
contact time (min)	X_1_	2	16	30
MOF dosage (g/L)	X_2_	0.1	0.55	1
pH	X_3_	4	7.5	11
ciprofloxacin (mg/L)	X_4_	30	62.5	100

**Table 4:** BBD design and removal levels for ciprofloxacin (CIP).

Run No.	Coded Variable				Response (% Removal)		Run No	Coded Variable				Response (% Removal)	
	X_1_	X_2_	X_3_	X_4_	Observed	Predicted		X_1_	X_2_	X_3_	X_4_	Observed	Predicted
1	−1	1	0	0	80	81	16	1	0	0	−1	97	100
2	−1	−1	0	0	73	68.2	17	−1	0	0	1	70	68.9
3	0	1	−1	0	79	79.7	18	0	0	0	0	94	96
4	1	0	1	0	75	73.6	19	0	1	0	1	79	79
5	0	1	1	0	65	62.7	20	1	0	−1	0	77	77.5
6	0	0	1	1	48	50.4	21	1	−1	0	0	96	92.5
7	0	−1	0	1	74	76.6	22	0	0	0	0	95	96
8	0	1	0	−1	98	96.2	23	0	0	1	−1	65	65.1
9	0	0	0	0	95	96	24	−1	0	−1	0	71	73.3
10	−1	0	1	0	41	41.1	25	−1	0	0	−1	76	78.4
11	1	1	0	0	91	93.4	26	0	0	−1	−1	86	81.2
12	0	−1	−1	0	70	73.9	27	0	0	−1	1	73	70.4
13	0	0	0	0	97	96	28	1	0	0	1	85	84
14	0	−1	1	0	54	54.8	29	0	−1	0	−1	84	84.9
15	0	0	0	0	99	96	-	-	-	-	-	-	-

**Table 5:** Estimated coefficients of the fitted polynomial model for CIP adsorption onto ZIF-67-SO_4_.

Model Term	Coefficient Estimate	Std. Error	*t*-Value	*p*-Value
Intercept	96.02	1.47	65.30	<0.0001
$X_{1}$	9.18	0.95	9.68	1.40 × 10^−7^
$X_{2}$	3.42	0.95	3.60	0.0028997
$X_{3}$	−9.03	0.95	−9.51	1.74 × 10^−7^
$X_{4}$	−6.38	0.95	−6.72	9.84 × 10^−6^
$X_{1}X_{2}$	−3.00	1.64	−1.82	0.0894262
$X_{1}X_{3}$	7.08	1.64	4.30	0.0007283
$X_{1}X_{4}$	−1.63	1.64	−0.99	0.3396952
$X_{2}X_{3}$	0.50	1.64	0.30	0.7654894
$X_{2}X_{4}$	−2.25	1.64	−1.37	0.1926667
$X_{3}X_{4}$	−1.00	1.64	−0.61	0.5527336
$X_{1}^{2}$	−6.82	1.29	−5.28	0.000116
$X_{2}^{2}$	−5.42	1.29	−4.20	0.0008955
$X_{3}^{2}$	−22.83	1.29	−17.69	5.66 × 10^−11^
$X_{4}^{2}$	−6.41	1.29	−4.96	0.0002087

**Table 6:** Optimum values for each independent variable.

Factor	Time (min)	MOF Dose (g/L)	pH	CIP (mg/L)	Removal (%)	
					Predicted	Experimental
Value	30	0.22	7.31	100	100	99.9

**Table 7:** The values of isotherm parameters.

Isotherm	Parameters	Values
Langmuir	b (L/mg)	1.89166
	q_e_ (mg/g)	2537.52777
	χ^2^	3024.69308
	SSE	12,098.77233
	$R_{\mathrm{Adj}}^{2}$	0.99567
Freundlich	K_f_ (mg/g)/(mg)^1/n^	1345.1812
	n	2.69053
	χ^2^	30,900.26595
	SSE	123,601.06379
	$R_{\mathrm{Adj}}^{2}$	0.9558
Jovanovic	q_m_ (mg·g^−1^)	2256.44168
	K_j_ (L·mg^−1^)	−1.64853
	χ^2^	4439.2249
	SSE	17,756.89959
	$R_{\mathrm{Adj}}^{2}$	0.99365
Temkin	A_T_ (L/mg)	31.62846
	b_T_	447.91592
	B (J/mol)	-
	χ^2^	15,237.21971
	SSE	60,948.87886
	$R_{\mathrm{Adj}}^{2}$	0.9782
Sips	q_ms_ (mg/g)	2593.86316
	K_S_ (L/mg)^ms^	1.71831
	m_s_	0.94708
	χ^2^	3786.66401
	SSE	11,359.99203
	$R_{\mathrm{Adj}}^{2}$	0.99458
Toth	K_T_	2577.35956
	A_T_	0.52871
	T_T_	0.94943
	χ^2^	3995.17545
	SSE	11,985.52634
	$R_{\mathrm{Adj}}^{2}$	0.99428
Khan	q_s_ (mg/g)	2772.45949
	b_K_	1.67977
	a_K_	1.03572
	χ^2^	3999.75536
	SSE	11,999.26609
	$R_{\mathrm{Adj}}^{2}$	0.99428

**Table 8:** Kinetic values of CIP adsorption onto ZIF.

Concentration (mg/L)	50	100	100
Agitation Type	Magnetic Stirrer		Sonication
q_e_, exp (mg/g)	256.4	509.06	560
Pseudo-First Order			
q_e_ (mg/g)	6.13223	480.42467	527.8629
k_1_ (min^−1^)	2.51783	2.58553	3.0163
χ^2^	156.19152	918.66385	1109.7541
SSE	780.95761	4593.31927	5548.7708
$R_{\mathrm{Adj}}^{2}$	0.93144	0.89479	0.86801
Pseudo-Second Order			
q_e_ (mg/g)	258.37326	514.80069	562.129
k_2_ (g/mg·min)	0.01483	0.0076	0.00849
χ^2^	16.21654	62.69378	65.9758
SSE	81.08269	313.4689	329.8794
$R_{\mathrm{Adj}}^{2}$	0.99288	0.99282	0.99215
Intraparticle Diffusion			
k_3_	40.94423	81.66703	80.3091
C	142.60978	285.45518	339.36581
χ^2^	618.10256	2061.48156	1950.1885
SSE	3090.51278	10,307.40782	9750.9426
$R_{\mathrm{Adj}}^{2}$	0.72867	0.76391	0.7680
Elovich			
a	10,708.00341	22,609.6118	56,005.9375
b	0.03048	0.0154	0.0156
χ^2^	214.18997	605.792	558.3242
SSE	1070.94984	3028.9600	2791.6209
$R_{\mathrm{Adj}}^{2}$	0.90598	0.9306	0.9335

**Table 9:** Thermodynamic parameters for CIP adsorption by ZIF-67-SO_4._

Temperature K	Ce mg/L	−∆G° kJ/mol	∆H° KJ/mol	∆S° KJ/mol.K
293	3.65	7.88	58.9	0.23
303	2.55	9.12		
313	0.7	12.88		
323	0.5	14.2		

## Supplementary Materials

The following are available online at https://www.mdpi.com/2079-4991/9/10/1422/s1, Table S1: Adequacy of the model tested, Table S2: Analysis of variance (ANOVA) for CIP removal by ZIF-67-SO4, Table S3: Nonlinear isotherm models and their parameters.
