# Supplementary material for: Enhanced Kinetic Removal of Ciprofloxacin onto Metal-Organic Frameworks by Sonication, Process Optimization and Metal Leaching Study
Source: Nanomaterials (Basel). 2019 Oct 8;9(10):1422. doi: 10.3390/nano9101422 (PMC6835299; doi:10.3390/nano9101422)
Supplement: Supplementary file 1 [file nanomaterials-09-01422-s001.pdf]

Supplementary

Enhanced kinetic removal of ciprofloxacin onto metal-organic frameworks by sonication, process optimization and metal leaching study

Synthesis of MOFs:

UIO-66: 0.2332 g of $ZrCl_4$ and 0.161 g terephthalic acid were dissolved in 50 mL DMF and stirred to obtain a clear solution. The solution then transferred to a 100 mL sealed Teflon autoclave and heated in an oven at 120ºC for 48 hours.

Cubic ZIF-8: In turn, 0.594 g $Zn(NO_3)_2{\cdot}6H_2O$ and 0.328 g (Hmim) added to 3 mL of distillated water and 3.76 g of NH_3 solution, separately. The zinc nitrate solution then added to Hmin solution and mixing continued for 10 minutes.

Dodecahedral ZIF-8: 0.863 g $Zn(OAc)_2$ and 11.350 g Hmim dissolved in 8 mL and 80 mL distilled water, respectively. After mixing the two solutions stirring continued for further 4 hours.

Leaf ZIF-8: 0.59 g of $Zn(NO_3)_2{\cdot}6H_2O$ and 1.3 g of Hmim added to 40 mL deionized water, separately. After the solution turn to clear, the two solutions were mixed for 4 h.

Cuboid ZIF-8: 1.3 g of Hmim dissolved in 40 ml distilled water containing 200 mg of Polyvinylpyrrolidone (PVP). Second solution was prepared by dissolving 0.595 g of $Zn(NO_3)_2{\cdot}6H_2O$ in distilled water. The two solutions then mixed for 30 min.

ZIF-67 with Granular and dodecahedron morphologies were prepared using $CoSO_4$, $Co(OAC)_2$, $CoCl_2$ and $Co(NO_3)_2$ salts, respectively. 1.642 g of Hmim and 1 mmol of cobalt salts were dissolved separately in 10 ml of deionized water. The solutions mixed and stirred for 30 min.

Table S1. Adequacy of the model tested.

Model formula	df	Sum Sq.	Mean Sq.	F value	Pr(>F)
First-order response	4	2617.2	654.3	60.526	0.00
Second order response	6	272	45.34	4.1941	0.01
pure quadratic response	4	3444.3	861.08	79.6545	0.00
Residuals	14	151.30	10.81		
Lack of fit (LOF)	10	136.1	13.61	3.57	0.1155
Pure error	4	15.2	3.81		

Multiple R-squared: 0.9767, Adjusted R-squared: 0.9533
F-statistic: 41.85 on 14 and 14 DF, p-value: 5.718e-09

Table S2. Analysis of variance (ANOVA) for CIP removal by ZIF-67-SO_4.

Source	Sum of Squares	df	Mean Square	F Value	p-value Prob > F
Model	6333.528	14	452.3949	41.84907	< 0.0001
X1-time	1012.003	1	1012.003	93.61599	< 0.0001
X2-MOF	140.0833	1	140.0833	12.95849	0.0029
X3-ph	977.4075	1	977.4075	90.41568	< 0.0001
X4-CIP	487.6875	1	487.6875	45.11383	< 0.0001
X1.X2	36	1	36	3.330202	0.0894
X1.X3	200.2225	1	200.2225	18.52171	0.0007
X1.X4	10.5625	1	10.5625	0.977091	0.3397
X2.X3	1	1	1	0.092506	0.7655
X2.X4	20.25	1	20.25	1.873239	0.1927
X2.X4	4	1	4	0.370022	0.5527
X1^2	301.5546	1	301.5546	27.89549	0.0001
X2^2	190.4325	1	190.4325	17.61607	0.0009
X3^2	3381.061	1	3381.061	312.7672	< 0.0001
X4^2	266.171	1	266.171	24.62231	0.0002
Residual	151.3422	14	10.81015		
Lack of Fit	136.1142	10	13.61142	3.575366	0.1155
Pure Error	15.228	4	3.807		
Cor Total	6484.870345	28			
Std. Dev.	3.29	R-Squared		0.9767	
Mean	78.86	Adj R-Squared		0.9533	
C.V. %	4.17	Pred R-Squared		0.8754	
PRESS	807.81	Adeq Precision		24.905	

Table S3. Nonlinear isotherm models and their parameters(24)

Isotherm	Equation	Parameters
Two-parameter models		
Langmuir	$q_e = \frac{Q_0 K_L C_e}{1 + K_L C_e}$	C_e= adsorbate equilibrium concentration (mg/L)<br>q_e = adsorption capacity at equilibrium (mg/g)<br>Q_0= monolayer coverage capacity (mg/g)<br>K_L= Langmuir isotherm constant (L/mg).
Freundlich	$q_e = K_f C_e^{1/n}$	K_f = Freundlich isotherm constant($mg^{1-(1/n)}$ $L^{1/n}$ g^{-1})<br>n = adsorption intensity
Jovanovic	$q_e = q_m\left(1 - e^{K_j C_e}\right)$	K_j = Jovanovic isotherm constant(L/mg)<br>q_m= maximum adsorption capacity(mg/g)
Temkin	$q_e = \frac{RT}{B} ln(A_T C_e)$	A_T = the Temkin isotherm equilibrium binding constant (L/mg)<br>b_T = the Temkin isotherm constant<br>R = the universal gas constant (8.314 J/mol K)<br>T = the temperature (K)<br>B = the constant related to heat of sorption (J/mol)
Dubinin–Radushkevich	$q_e = Q_m exp(-K\varepsilon^2)$	K = Dubinin–Radushkevich isotherm constant (mol^2/kJ^2)<br>Q_m = is the theoretical isotherm saturation capacity (mg/g)<br>$\boldsymbol{\varepsilon}$ = is the Polanyi potential
Three-parameter models		

Hill	$q_e = \frac{q_{s_H} C_e^{n_H}}{K_D + C_e^{n_H}}$	q_{s_H}= Hill isotherm maximum uptake saturation (mg/L)<br><br>K_D= Hill constant<br><br>n_H= Hill cooperativity coefficient of the binding interaction.
Sips	$q_e = \frac{q_{m_s} K_s C_e^{m_s}}{1 + K_s C_e^{m_s}}$	q_{m_s}= Sips maximum adsorption capacity (mg/g)<br><br>K_S = Sips equilibrium constant $(L/mg)^{m_s}$<br><br>m_S= Sips model exponent
Koble-Corrigan	$q_e = \frac{a C_e^n}{1 + b C_e^n}$	a = Koble-Corrigan parameter($L^n mg^{1-n}/g$)<br><br>b = Koble-Corrigan parameter$(L/mg)^n$<br><br>n = Koble-Corrigan parameter
Toth	$q_e = \frac{K_T C_e}{\left[A_T + C_e^{T_T}\right]^{1/T_T}}$	K_T= Toth equilibrium constant<br><br>T_T= Toth model exponent
Redlich-Peterson	$q_e = \frac{K_{RP} C_e}{1 + a_{RP} C_e^g}$	K_{RP} = Redlich-Peterson isotherm constant(L/g)<br><br>a_{RP}=Redlich-Peterson model constant$(mg/L)^{-g}$<br><br>g = Redlich-Peterson model exponent
Khan	$q_e = \frac{q_s b_K C_e}{(1 + b_K C_e)^{n_K}}$	b_K= Khan isotherm constant<br><br>q_s= theoretical isotherm saturation capacity(mg/g)<br><br>n_K= Khan model exponent
